# A Novel 2-D Coherent DOA Estimation Method Based on Dimension Reduction Sparse Reconstruction for Orthogonal Arrays

**DOI:** 10.3390/s16091496

**Published:** 2016-09-15

**Authors:** Xiuhong Wang, Xingpeng Mao, Yiming Wang, Naitong Zhang, Bo Li

**Affiliations:** 1 School of Electronics and Information Engineering, Harbin Institute of Technology, Harbin 150001, China; xiuhongwang@hit.edu.cn (X.W.); wym.fio@gmail.com (Y.W.); ntzhang@hit.edu.cn (N.Z.); 2 School of Information and Electrical Engineering, Harbin Institute of Technology (Weihai), Weihai 264209, China; libo1983@hit.edu.cn; 3 First Institute of Oceanography, SOA, Qingdao 266061, China; 4 Collaborative Innovation Center of Information Sensing and Understanding at Harbin Institute of Technology, Harbin 150001, China

**Keywords:** direction of arrival (DOA), two-dimensional (2-D) DOA estimation, coherent sources, dimension reduction sparse reconstruction, redundant sub-dictionary

## Abstract

Based on sparse representations, the problem of two-dimensional (2-D) direction of arrival (DOA) estimation is addressed in this paper. A novel sparse 2-D DOA estimation method, called Dimension Reduction Sparse Reconstruction (DRSR), is proposed with pairing by Spatial Spectrum Reconstruction of Sub-Dictionary (SSRSD). By utilizing the angle decoupling method, which transforms a 2-D estimation into two independent one-dimensional (1-D) estimations, the high computational complexity induced by a large 2-D redundant dictionary is greatly reduced. Furthermore, a new angle matching scheme, SSRSD, which is less sensitive to the sparse reconstruction error with higher pair-matching probability, is introduced. The proposed method can be applied to any type of orthogonal array without requirement of a large number of snapshots and a priori knowledge of the number of signals. The theoretical analyses and simulation results show that the DRSR-SSRSD method performs well for coherent signals, which performance approaches Cramer–Rao bound (CRB), even under a single snapshot and low signal-to-noise ratio (SNR) condition.

## 1. Introduction

Direction of Arrival (DOA) estimation methods, especially those of high accuracy and high resolution, have been rapidly developed in recent years, many of which can be found in applications of radar, sonar, communications, etc. [1]. While most of those methods are for one dimensional (1-D) direction (e.g., azimuth angle), during estimation of multiple plane waves, in many applications such as ground target observation of airborne or space borne radar, it is more reasonable to assume that the arriving signals have the two-dimensional (2-D) (i.e., azimuth and elevation angles) nature. Therefore, extensive attention and in-depth research are given to deal with the 2-D DOA estimation problem [2,3,4,5].

The classical subspace-based methods [6,7,8], such as 2-D Multiple Signal Classification (2-D MUSIC) [6], 2-D Estimate Signal Parameter via Rotational Invariance Technique (2-D ESPRIT) [7], cross-correlation based 2-D DOAs estimation (CODE) [8], etc., have been successfully applied in 2-D DOA estimation with high accuracy and resolution under the strict constrains of large snapshots, independent signals, uncorrelated white noise, etc. However, these subspace based methods will lose efficacy in practical applications where the target signal immerged into strong correlated sources and noises after propagated multiple paths and experienced complex electromagnetic interference. Therefore, it is of importance to suppress the influence of correlated sources and quickly estimate 2-D DOA under the practical environment and complex interferences. Some progresses have been made in this direction [9,10,11,12,13,14,15,16,17,18,19,20,21,22,23,24]. The first class of approach is spatial smoothing based method. The spatial smoothing (SS) [9,10] was first applied to the l-shaped array for signal decoherence and followed by estimation using ESPRIT method in [11]. Furthermore, the spatial smoothing method was applied to circular array in [12]. The second class of approach is data matrix reconstruction based method, which facilitated decoherence by matrix reconstruction in l-shaped array, double parallel linear array, sparse uniform rectangular plane array and uniform matrix array [13,14,15]. The 2-D DOA estimation was then realized by classical subspace based methods such as ESPRIT or Propagator Method (PM). The third class of approach is Toeplitz matrix construction based method, which signal decoherence was realized by either building the received array data directly into a Toeplitz matrix structure [16,17] or constructing fourth-order cumulant Toeplitz matrix to cope with the color noise [18]. Furthermore, there are some particular approaches, such as in [19], where the oblique projection method was exploited to separate the coherent and incoherent signals with two parallel uniform linear arrays, which was followed by 2-D DOA estimation of all signals. By decoupling angles, Wong et al. [20,21,22,23,24] convert a 2-D DOA estimation into two 1-D DOA estimation. The work in [21,22] focus on the unambiguous 2-D DOA estimation on a sparse planar array with dual/multiple scales of invariance and the work in [23,24] focus on the 2-D DOA estimation on uniform l-shaped array. In 1-D DOA estimation, the estimation methods by Zoltowski et al. [21,22] and by Wong [23,24] are based on ESPRIT and Root-MUSIC algorithm, respectively. Their pairing methods are based on a comparison of eigenvalues of the covariance matrix or a related processing of noise subspace. Therefore, these methods have the same drawbacks as subspace-based methods, such as requiring a large number of snapshots, non-coherent signals, etc.

Compressed Sensing (CS), which uses the ideas of sparse decomposition, has been applied in the DOA estimation [25,26,27,28,29,30,31] by building the angle redundant dictionary and solving the optimal sparse solution. It achieves high accuracy and high resolution. Furthermore, the preprocessing of spatial smoothing and statistical property of the received data are not required. Therefore DOA estimation can be realized without a large amount of snapshots which has a great potential in application. However, it has not been applied in the 2-D DOA estimation due to the long redundant dictionary and high complexity, and most applications can only be found in 1-D DOA estimation [25,26] of uniform linear array (ULA). In [27], the 2-D DOA estimation was transformed into two 1-D estimation by exploiting the geometry of l-shaped array, then the method based on sparse representation of space angle with amplitude-comparison pairing (SRSA-AC) is proposed, but its pairing algorithm would fail in close or equal signal powers.

To solve the problem of 2-D coherent DOA estimation in low complexity, a novel Dimension Reduction Sparse Reconstruction (DRSR) with pair-matching by Spatial Spectrum Reconstruction of Sub-Dictionary (SSRSD) method is proposed based on the two orthogonal linear arrays. The computational complexity is greatly reduced because the 2-D redundant dictionary is converted to two independent 1-D redundant dictionaries by exploring the structure feature of orthogonal linear arrays. Moreover, the matching probability and estimation speed are improved.

The advantages of the proposed method are mainly in the following aspects respects: (1) the coherent DOA estimation can be executed directly without decoherence process such as spatial smoothing; (2) the computational complexity and execution time are greatly reduced by transforming the 2-D DOA estimation into parallel computations of two independent 1-D DOAs; (3) the requirement of large number of snapshots is relaxed such that the 2-D DOA estimation performs well in any number of snapshots; and (4) prior knowledge of the number of signals is no longer a requirement.

The rest of this paper is organized as follows: firstly, signal model and 2-D DOA estimation based on direct sparse representation (DSR) are introduced in Section 2. Secondly, a novel DRSR method for 2-D DOA estimation is proposed in Section 3. A new pair-matching scheme based on SSRSD is proposed in Section 4. Then, the CRB and the computational complexity of the proposed method are analyzed in Section 5. The simulation results and some discussions are given in Section 6. Finally, the conclusion of our work is summarized in Section 7.

## 2. Signal Model and 2-D DOA Estimation Based on Direct Sparse Representation

### 2.1. Signal Model

**Figure 1:**
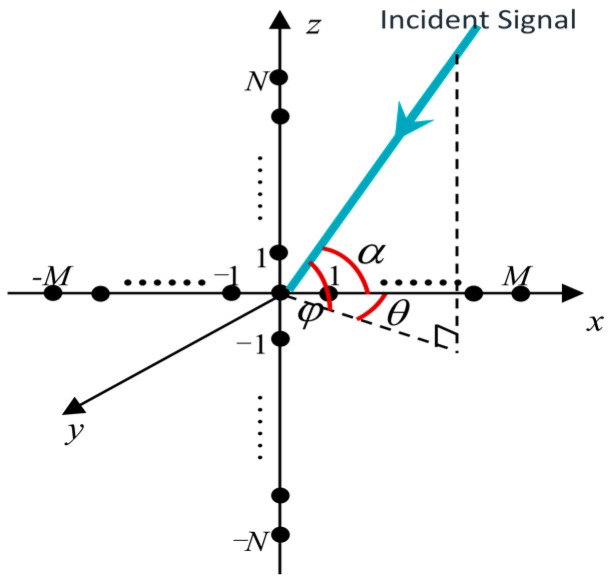
Cross array configuration for 2-D DOA estimation.

Consider K far-field narrowband signals impinging on a centrosymmetric uniform cross array in the *x-z* plane as shown in Figure 1, where the subarray along *x-*axis has 2M+1 omnidirectional sensors with spacing $d_{x}$ and the other subarray along *z-*axis has 2N+1 omnidirectional sensors with spacing $d_{z}$. Let $\theta_{k}$ and $\varphi_{k}$ be the azimuth and elevation angles of the *k*th signal, respectively. Note that the azimuth angle is taken between the signal arrival direction and *x-*axis, and the elevation angle is taken between signal arrival direction and *z-*axis.

The received data of the *m*th array element at time t is expressed as

(1) $$r_{m}\left(t\right)=\sum_{k=1}^{K}a_{m}\left(\theta_{k},\varphi_{k}\right)s_{k}\left(t\right)+e_{m}\left(t\right)$$

where $s_{k}\left(t\right)$ is the *k*th incident signal, $e_{m}\left(t\right)$ is the additive noise of the *m*th element, and $a_{m}\left(\theta_{k},\varphi_{k}\right)$ is the steering vector of the array and is represented by

(2) $$a_{m}\left(\theta_{k},\varphi_{k}\right)=\exp\left(-j\frac{2\pi }{\lambda }\left(x_{m}\cos\theta_{k}\cos\varphi_{k}+z_{m}\sin\varphi_{k}\right)\right),\;m=1,2,\cdots ,M_{c}$$

where $x_{m}$ and $z_{m}$ denote the coordinate position of the *m*th element in the *xoz* plane, and $M_{c}=2M+2N+1$ gives the total number of elements.

For all received elements, the received data can be represented by

(3) R(t)=AS(t)+E(t)

where $R\left(t\right)={\left[r_{1}\left(t\right),r_{2}\left(t\right),\cdots ,r_{M_{c}}\left(t\right)\right]}^{T}\in {\mathbb{C}}^{M_{c}\times 1}$ and $E\left(t\right)={\left[e_{1}\left(t\right),e_{2}\left(t\right),\cdots ,e_{M_{c}}\left(t\right)\right]}^{T}\in {\mathbb{C}}^{M_{c}\times 1}$ are the received signal and the noise of all array elements, respectively; $S\left(t\right)={\left[s_{1}\left(t\right),\cdots ,s_{K}\left(t\right)\right]}^{T}\in {\mathbb{C}}^{K\times 1}$ is incident signal of K sources; and $A\in {\mathbb{C}}^{M_{c}\times K}$ is the manifold matrix of the array given by

(4) $$A=\left[a\left(\theta_{1},\varphi_{1}\right),a\left(\theta_{2},\varphi_{2}\right),\cdots ,a\left(\theta_{K},\varphi_{K}\right)\right]$$

where $a\left(\theta_{k},\varphi_{k}\right)={\left[a_{1}\left(\theta_{k},\varphi_{k}\right),a_{2}\left(\theta_{k},\varphi_{k}\right),\cdots ,a_{M_{c}}\left(\theta_{k},\varphi_{k}\right)\right]}^{T}$ is the steering vector corresponding to 2-D angle pair of $\left(\theta_{k},\varphi_{k}\right)$.

### 2.2. 2-D DOA Estimation Based on Direct Sparse Representation (DSR)

#### 2.2.1. Single Snapshot Estimation

The model of array received signal in Equation (3) can be converted into sparse representation model based on the sparse decomposition. The spatial domain is equally divided into respective azimuth and elevation angle sets of $\bar{\Theta }=\left[{\bar{\theta }}_{1},{\bar{\theta }}_{2},\cdots ,{\bar{\theta }}_{L_{\theta }}\right]$ and $\bar{\Xi }=\left[{\bar{\varphi }}_{1},{\bar{\varphi }}_{2},\cdots ,{\bar{\varphi }}_{L_{\varphi }}\right]$ for searching, where $L_{\theta }$ and $L_{\varphi }$ are the number of angles. The incident signal S(t) is sparsely represented in spatial domain as $P\left(t\right)\in {\mathbb{C}}^{L\times 1}$, where $p_{i}\left(t\right),\left(i=1,2,\cdots ,L\right)$ denotes the complex amplitude of signal at the potential incident angle, whose total number is $L≜L_{\theta }L_{\varphi }$. Therefore, we have

(5) $${\left\|P\left(t\right)\right\|}_{0}=\left|\mathrm{supp}\left(P\right)\right|=K$$

where supp(P) denotes a set of indices of non-zero elements of vector P(t).

The angle redundant dictionary $\Psi \in {\mathbb{C}}^{M_{c}\times L}$ is then created by extending the columns of the array manifold matrix in Equation (3). It consists of steering vectors of the 2-D spatial angles, and is written as

(6) $$\Psi \left(\bar{\Theta },\bar{\Xi }\right)=\left\{a\left(\theta ,\varphi \right)|\theta \in \bar{\Theta },\varphi \in \bar{\Xi }\right\}=\left[a\left({\bar{\theta }}_{1},{\bar{\varphi }}_{1}\right),a\left({\bar{\theta }}_{1},{\bar{\varphi }}_{2}\right),\cdots ,a\left({\bar{\theta }}_{L_{\theta }},{\bar{\varphi }}_{L_{\varphi }}\right)\right]$$

Therefore, the sparse representation of the received signal in 2-D DOA estimation is

(7) $$R\left(t\right)=\Psi \left(\bar{\Theta },\bar{\Xi }\right)P\left(t\right)+E\left(t\right)$$

The DOA estimation in Equation (3) is then transformed into the sparse recovery problem in Equation (7), and the solution can be obtained by

(8) $$\min{\left\|P\left(t\right)\right\|}_{1}\;s.t.\;R\left(t\right)=\Psi \left(\bar{\Theta },\bar{\Xi }\right)P\left(t\right)+E\left(t\right)$$

The constrained minimization problem in Equation (8) can be alternatively solved by convex optimization as

(9) $$\hat{P}\left(t\right)=\arg\underset{P\left(t\right)}{\min}{\left\|R\left(t\right)-\Psi \left(\bar{\Theta },\bar{\Xi }\right)P\left(t\right)\right\|}_{2}^{2}+\mu {\left\|P\left(t\right)\right\|}_{1}$$

where ${\left\|\cdot \right\|}_{2}$ is the *l*_2_ norm, ${\left\|\cdot \right\|}_{1}$ is the *l*_1_ norm, μ>0 is regularization parameter which balances the relation between Frobenius norm and *l*_1_ norm, where the larger μ indicates the solution approaches closer to zero vector and the smaller μ indicates the solution approximates to the least squares solution. The convex optimization in Equation (9) is a Second-Order Cone Programming (SOCP), which can be solved by conic optimization software such as SeDuMi or CVX.

#### 2.2.2. Multiple Snapshots Estimation

The multiple snapshots scenario can be obtained by extending the single snapshot as

(10) $$R=\Psi \left(\bar{\Theta },\bar{\Xi }\right)P+E$$

where $R=[R\left(t_{1}\right),R\left(t_{2}\right),\cdots ,R\left(t_{J}\right)]\in {\mathbb{C}}^{M_{c}\times J}$ and $P=[P\left(t_{1}\right),P\left(t_{2}\right),\cdots ,P\left(t_{J}\right)]\in {\mathbb{C}}^{L\times J}$ denote a number of *J* snapshots of respective received and incident signals by sensor array, respectively; and $E=[E\left(t_{1}\right),E\left(t_{2}\right),\cdots ,E\left(t_{J}\right)]\in {\mathbb{C}}^{M_{c}\times J}$ is additive white noise.

The sparse solution of (10) can be solved by

(11) $$\hat{P}=\arg\underset{P}{\min}{\left\|R-\Psi \left(\bar{\Theta },\bar{\Xi }\right)P\right\|}_{F}^{2}+\mu {\left\|P\right\|}_{2,1}$$

where ${\left\|\cdot \right\|}_{F}$ is the Frobenius norm, ${\left\|P\right\|}_{2,1}$ is the mixed *l*_2,1_ norm of matrix P and is defined as ${\left\|P\right\|}_{2,1}≜\sum_{l=1}^{L}{\left\|P(l,:)\right\|}_{2}=\sum_{l=1}^{L}\sqrt{\sum_{j=1}^{J}{\left(P(l,j)\right)}^{2}}$.

Define the normalized spatial spectrum in sparse decomposition framework as

(12) $$Q\left(\theta ,\varphi \right)=\mathrm{unvec}\left(\frac{P_{v}}{\max\left\{P_{v}\right\}}\right)$$

where $P_{v}\left(l\right)≜{\left\|\hat{P}(l,:)\right\|}_{2},\;l=1,2,...,L$, unvec(·) indicates converting vector into matrix.

Herein, the DOA estimation can be written as

(13) $$\left(\hat{\theta },\hat{\varphi }\right)=\arg\underset{\left(\theta ,\varphi \right)}{\max}\;Q\left(\theta ,\varphi \right)$$

It can be observed that the key of sparse representation based 2-D DOA estimation lies in the sparse solution of P, where the complexity is decided by the scale of P and the dimension $L=L_{\theta }L_{\varphi }$ in constructing redundant dictionary $\Psi \left(\bar{\Theta },\bar{\Xi }\right)$. The dimension of redundant dictionary could be enormous when the number of azimuth and elevation searing points is large, while the increasing dimension will lead to a sharp increase in the computational complexity of sparse solution in Equations (9) and (11), or even become unachievable.

## 3. 2-D DOA Estimation by Dimension Reduction Sparse Reconstruction

To reduce the computational complexity of 2-D DOA estimation, curtailing the scale or dimension of the redundant dictionary can be considered. Therefore, the 2-D DOA estimation can be divided into two independent 1-D DOA estimations through angle decoupling, thus the dimension of redundant dictionary is greatly decreased.

Considering the cross array as two independent ULAs, it can be observed from Figure 1 that the included angle between incident signal and the subarray along *z-*axis is π/2−φ. Therefore, the elevation angle φ can be estimated by the *z-*axis subarray. Similarly, the included angle (also called synthesis azimuth angle, written as α) between incident signal and the subarray along *x*-axis can be estimated by the *x*-axis subarray. Note that there is an important relationship between azimuth angle θ and elevation angle φ as

(14) cosα=cosθcosφ

Therefore the azimuth angle θ can be determined from Equation (14).

### 3.1. Single Snapshot Estimation

The signals received by the subarray along the *z-*axis and the *x*-axis are represented as

(15) $$\begin{array}{l} R_{Z}\left(t\right)=A_{Z}S\left(t\right)+E_{Z}\left(t\right)\\ R_{X}\left(t\right)=A_{X}S\left(t\right)+E_{X}\left(t\right) \end{array}$$

where $A_{Z}=\left[a_{Z}\left(\varphi_{1}\right),\ldots ,a_{Z}\left(\varphi_{K}\right)\right]\in {\mathbb{C}}^{\left(2N+1\right)\times K}$ and $A_{X}=\left[a_{X}\left(\alpha_{1}\right),\ldots ,a_{X}\left(\alpha_{K}\right)\right]\in {\mathbb{C}}^{\left(2M+1\right)\times K}$ denote the respective subarray manifolds, and

(16) $$a_{Z}\left(\varphi \right)={\left[\begin{array}{ccccc} e^{j\frac{2\pi }{\lambda }Nd_{z}\sin\varphi } & e^{j\frac{2\pi }{\lambda }\left(N-1\right)d_{z}\sin\varphi } & \cdots \;1\;\cdots & e^{-j\frac{2\pi }{\lambda }\left(N-1\right)d_{z}\sin\varphi } & e^{-j\frac{2\pi }{\lambda }Nd_{z}\sin\varphi } \end{array}\right]}^{T}$$

(17) $$a_{X}\left(\alpha \right)={\left[\begin{array}{ccccc} e^{j\frac{2\pi }{\lambda }Md_{x}\cos\alpha } & e^{j\frac{2\pi }{\lambda }\left(M-1\right)d_{x}\cos\alpha } & \cdots \;1\;\cdots & e^{-j\frac{2\pi }{\lambda }\left(M-1\right)d_{x}\cos\alpha } & e^{-j\frac{2\pi }{\lambda }Md_{x}\cos\alpha } \end{array}\right]}^{T}$$

The sparse representation of Equation (15) is

(18) $$\begin{array}{l} R_{Z}\left(t\right)=\Phi_{Z}\left(\bar{\Xi }\right)P_{Z}\left(t\right)+E_{Z}\left(t\right)\\ R_{X}\left(t\right)=\Phi_{X}\left(\bar{\Lambda }\right)P_{X}\left(t\right)+E_{X}\left(t\right) \end{array}$$

where $\Phi_{Z}\left(\bar{\Xi }\right)\in {\mathbb{C}}^{\left(2N+1\right)\times L_{\varphi }}$ and $\Phi_{X}\left(\bar{\Lambda }\right)\in {\mathbb{C}}^{\left(2M+1\right)\times L_{\theta }}$ denote the redundant dictionaries of the corresponding subarrays formed by the expansion of $A_{Z}$ and $A_{X}$, and are represented as

(19) $$\Phi_{Z}\left(\bar{\Xi }\right)=\left[\begin{array}{cccc} a_{Z}\left({\bar{\varphi }}_{1}\right) & a_{Z}\left({\bar{\varphi }}_{2}\right) & \cdots & a_{Z}\left({\bar{\varphi }}_{L_{\varphi }}\right) \end{array}\right]$$

(20) $$\Phi_{X}\left(\bar{\Lambda }\right)=\left[\begin{array}{cccc} a_{X}\left({\bar{\alpha }}_{1}\right) & a_{X}\left({\bar{\alpha }}_{2}\right) & \cdots & a_{X}\left({\bar{\alpha }}_{L_{\theta }}\right) \end{array}\right]$$

where $\bar{\Xi }=\left[{\bar{\varphi }}_{1},{\bar{\varphi }}_{2},\cdots ,{\bar{\varphi }}_{L_{\varphi }}\right]$ and $\bar{\Lambda }=\left[{\bar{\alpha }}_{1},{\bar{\alpha }}_{2},\cdots ,{\bar{\alpha }}_{L_{\theta }}\right]$ are the sets of elevation or synthesis azimuth angles, respectively.

Therefore, the DOA estimation in Equation (18) can be solved as

(21) $$\begin{array}{l} {\hat{P}}_{Z}=\arg\underset{P_{Z}}{\min}{\left\|R_{Z}\left(t\right)-\Phi_{Z}\left(\bar{\Xi }\right)P_{Z}\left(t\right)\right\|}_{2}+\mu_{1}{\left\|P_{Z}\left(t\right)\right\|}_{1}\\ {\hat{P}}_{X}=\arg\underset{P_{X}}{\min}{\left\|R_{X}\left(t\right)-\Phi_{X}\left(\bar{\Lambda }\right)P_{X}\left(t\right)\right\|}_{2}+\mu_{2}{\left\|P_{X}\left(t\right)\right\|}_{1} \end{array}$$

### 3.2. Multiple Snapshots Estimation

By extending the single snapshot case in Equation (15), the sparse representation in multiple snapshots is obtained as

(22) $$R_{Z}=\Phi_{Z}\left(\bar{\Xi }\right)P_{Z}+E_{Z}\;R_{X}=\Phi_{X}\left(\bar{\Lambda }\right)P_{X}+E_{X}$$

where $R_{Z}=\left[R_{Z}\left(t_{1}\right),\cdots ,R_{Z}\left(t_{J}\right)\right]$, $P_{Z}=\left[P_{Z}\left(t_{1}\right),\cdots ,P_{Z}\left(t_{J}\right)\right]$, $R_{X}=\left[R_{X}\left(t_{1}\right),\cdots ,R_{X}\left(t_{J}\right)\right]$, and $P_{X}=\left[P_{X}\left(t_{1}\right),\cdots ,P_{X}\left(t_{J}\right)\right]$ denote a number of *J* snapshots of respective received or incident signals by the subarrays along *z* axis or *x* axis.

To reduce the computational complexity in solving the solution when the number of snapshots increases, singular value decomposition(SVD) of $R_{Z}$ and $R_{X}$ can be performed as

(23) $$R_{Z}=U_{Z}\Lambda_{Z}V_{Z}^{H}\;R_{X}=U_{X}\Lambda_{X}V_{X}^{H}$$

Define matrix $D_{K}={\left[I_{K},0_{K\times \left(J-K\right)}\right]}^{T}$ and generate the data vectors after dimension reduction as

(24) $$R_{Z}^{\mathrm{SVD}}=R_{Z}V_{Z}D_{K};\;R_{X}^{\mathrm{SVD}}=R_{X}V_{X}D_{K}$$

Similarly, $S_{Z}^{\mathrm{SVD}}=P_{Z}V_{Z}D_{K}$, $S_{X}^{\mathrm{SVD}}=P_{X}V_{X}D_{K}$, $E_{Z}^{\mathrm{SVD}}=E_{Z}V_{Z}D_{K}$, $E_{X}^{\mathrm{SVD}}=E_{X}V_{X}D_{K}$ can be represented and Equation (22) can be reconstructed as the DOA estimation model as

(25) $$R_{Z}^{\mathrm{SVD}}=\Phi_{Z}S_{Z}^{\mathrm{SVD}}+E_{Z}^{\mathrm{SVD}};\;R_{X}^{\mathrm{SVD}}=\Phi_{X}S_{X}^{\mathrm{SVD}}+E_{X}^{\mathrm{SVD}}$$

The sparse solution of Equation (22) can be solved by

(26) $$\begin{array}{l} {\hat{S}}_{Z}=\arg\underset{S_{Z}}{\min}{\left\|R_{Z}^{\mathrm{SVD}}-\Phi_{Z}\left(\bar{\Xi }\right)S_{Z}^{\mathrm{SVD}}\right\|}_{F}+\mu_{1}{\left\|S_{Z}^{\mathrm{SVD}}\right\|}_{2,1}\\ {\hat{S}}_{X}=\arg\underset{S_{X}}{\min}{\left\|R_{X}^{\mathrm{SVD}}-\Phi_{X}\left(\bar{\Lambda }\right)S_{X}^{\mathrm{SVD}}\right\|}_{F}+\mu_{2}{\left\|S_{X}^{\mathrm{SVD}}\right\|}_{2,1} \end{array}$$

Therefore, the 1-D spatial spectrums of $Q_{Z}\left(\varphi \right)$ and $Q_{X}\left(\alpha \right)$ are solved by

(27) $$\begin{array}{l} Q_{Z}\left({\bar{\varphi }}_{l}\right)={\left\|{\hat{S}}_{Z}(l,:)\right\|}_{2}/\max\left\{{\left\|{\hat{S}}_{Z}(l,:)\right\|}_{2}\right\},\;l=1,2,\cdots ,L_{\varphi }\\ Q_{X}\left({\bar{\alpha }}_{l}\right)={\left\|{\hat{S}}_{X}(l,:)\right\|}_{2}/\max\left\{{\left\|{\hat{S}}_{X}(l,:)\right\|}_{2}\right\},\;l=1,2,\cdots ,L_{\theta } \end{array}$$

The elevation and synthesis angles corresponding to a number of *K* largest peaks in the spatial spectrum can be estimated as

(28) $$\hat{\varphi }=\arg\underset{\varphi }{\max}\;Q_{Z}\left(\bar{\varphi }\right)\;\hat{\alpha }=\arg\underset{\alpha }{\max}\;Q_{X}\left(\bar{\alpha }\right)$$

## 4. Angle Pair-Matching Scheme Based on Sub-Dictionary Spatial Spectrum Reconstruction

Each elevation angle ${\hat{\varphi }}_{k}$ or synthesis angle ${\hat{\alpha }}_{k}$ is estimated separately in Section 3 and we need an angle pairing scheme to pair them. Thereby, the corresponding azimuth angle can be calculated as

(29) $${\hat{\theta }}_{k}=\arccos\left(\frac{\cos\alpha_{k}}{\cos\varphi_{k}}\right)$$

Consequently, the 2-D DOA is obtained as $\left({\hat{\theta }}_{k},{\hat{\varphi }}_{k}\right)$.

In [27], the angle pairing scheme is based on the comparison of estimated amplitudes of each incident signal, which means the nonzero elements of the estimated amplitudes ${\hat{S}}_{Z}$ and ${\hat{S}}_{X}$ are sorted and one-to-one correspondence is established. However, it is under the premise of all amplitudes are correctly estimated, which requires high-precision reconstruction algorithm, high SNR as well as certain amplitude difference between the target signals. Therefore, the amplitude sequencing error will lead to incorrect angle matching if the amplitudes estimation are not correct. Furthermore, that angle pairing scheme is not effective when the signal powers are equal or close.

The choice of parameters $\mu_{1}$ and $\mu_{2}$ in solving Equation (26) would have great influence on amplitude estimation error and pair-matching probability. In Equation (26), the Frobenius-norm term represents the joint estimation error, which connects to the amplitude estimation error and position locating error (i.e., DOA estimation error), whereas the *l*_1_-norm term is the sparsity guarantees. The parameter μ balances the contributions of each estimation errors. To assure a maximized pair-matching probability, the regularization parameter selected in [27] need to guarantee that amplitude estimation error contribute a higher proportion. Thus, the lower proportion of position locating error would lead to a larger DOA estimation error.

To increase both the pair-matching probability and DOA estimation performance, a new angle pairing scheme based on spatial spectrum of redundant sub-dictionary is proposed. Firstly, the redundant sub-dictionary is constructed. It is assumed that there are K incident signals and their elevation angles $\left\{\varphi_{1},\cdots ,\varphi_{K}\right\}$ and synthesis angles $\left\{\alpha_{1},\cdots ,\alpha_{K}\right\}$ are estimated in Section 3. Thus, there are a total of $I=K^{2}$ possible pairs of 2-D angles, which can be represented as

(30) $$\Sigma =\left\{\left(\hat{\theta },\hat{\varphi }\right)|\left({\hat{\theta }}^{\;i},{\hat{\varphi }}^{i}\right),i=1,2,\cdots ,I\right\}$$

where ${\hat{\varphi }}^{i}$ is obtained by Equation (29). 

The 2-D rectangular region of angles is formed by extending the radiuses along elevation and azimuth direction centered at $\left({\hat{\theta }}^{\;i},{\hat{\varphi }}^{i}\right)$, represented as

(31) $$\prod_{i}=\left\{\left(\theta ,\varphi \right)|\theta \in \left({\hat{\theta }}^{\;i}-r_{\theta },{\hat{\theta }}^{\;i}+r_{\theta }\right),\varphi \in \left({\hat{\varphi }}^{i}-r_{\varphi },{\hat{\varphi }}^{i}+r_{\varphi }\right)\right\}$$

In region of $\prod_{i}$, the angles are extracted uniformly by the respective intervals of $\Delta_{\theta }$ and $\Delta_{\varphi }$, denoted as

(32) $$\Omega_{i}=\left\{\left(\theta ,\varphi \right)|\begin{array}{cc} \theta ={\hat{\theta }}^{i}+j\Delta_{\theta }, & j=-\left\lfloor r_{\theta }/\Delta_{\theta }\right\rfloor ,\cdots ,0,\cdots ,\left\lfloor r_{\theta }/\Delta_{\theta }\right\rfloor \\ \varphi ={\hat{\varphi }}^{i}+l\Delta_{\varphi }, & l=-\left\lfloor r_{\varphi }/\Delta_{\varphi }\right\rfloor ,\cdots ,0,\cdots ,\left\lfloor r_{\varphi }/\Delta_{\varphi }\right\rfloor \end{array}\right\}$$

The steering vector $g_{i}=a\left(\theta ,\varphi \right),\left(\theta ,\varphi \right)\in \Omega_{i}$ is generated according to Equation (4) by all angles in $\Omega_{i}$, then forms a set as

(33) $$G_{i}\left(\Omega_{i}\right)=\left[g_{1}^{i},g_{2}^{i},\cdots ,g_{T}^{i}\right],\;T=\left(2\left\lfloor r_{\theta }/\Delta_{\theta }\right\rfloor +1\right)\times \left(2\left\lfloor r_{\varphi }/\Delta_{\varphi }\right\rfloor +1\right)$$

Set $G^{i}$ as the redundant sub-dictionary centered at $\left({\hat{\theta }}^{\;i},{\hat{\varphi }}^{i}\right)$, and write the combination set of a number of I sub-dictionaries as

(34) $$G=\{\begin{array}{cc} \left[G\left(\Omega_{1}\right),G\left(\Omega_{2}\right),\cdots ,G\left(\Omega_{I}\right)\right] & if\;\Omega_{1}\cap \Omega_{2}\cap \cdots \cap \Omega_{I}=\emptyset \\ G\left(\Omega_{1}\cup \Omega_{2}\cup \cdots \cup \Omega_{I}\right) & if\;\Omega_{1}\cap \Omega_{2}\cap \cdots \cap \Omega_{I}\neq \emptyset \end{array}$$

As each sub-dictionary is a part of the complete dictionary, they still satisfy the DOA sparse representation model of the received signals in Equation (7) or (10). Hence,

(35) R=GB+E

Furthermore, the normalized 2-D spatial spectrum under redundant sub-dictionaries is defined as

(36) $$Q\left(\theta ,\varphi \right)=\mathrm{unvec}\left({\hat{B}}_{v}/\max\left\{{\hat{B}}_{v}\right\}\right)$$

where ${\hat{B}}_{v}\left(i\right)={\left\|\hat{B}(i,:)\right\|}_{2},\;i=1,2,...,I\times T$, $\hat{B}$ is the sparse solution of Equation (35), and can be solved by

(37) $$\begin{array}{ccc} \min{\left\|B\right\|}_{2,1} & s.t. & {\left\|R-GB\right\|}_{F}^{2}\leq \beta \end{array}$$

**Figure 2:**
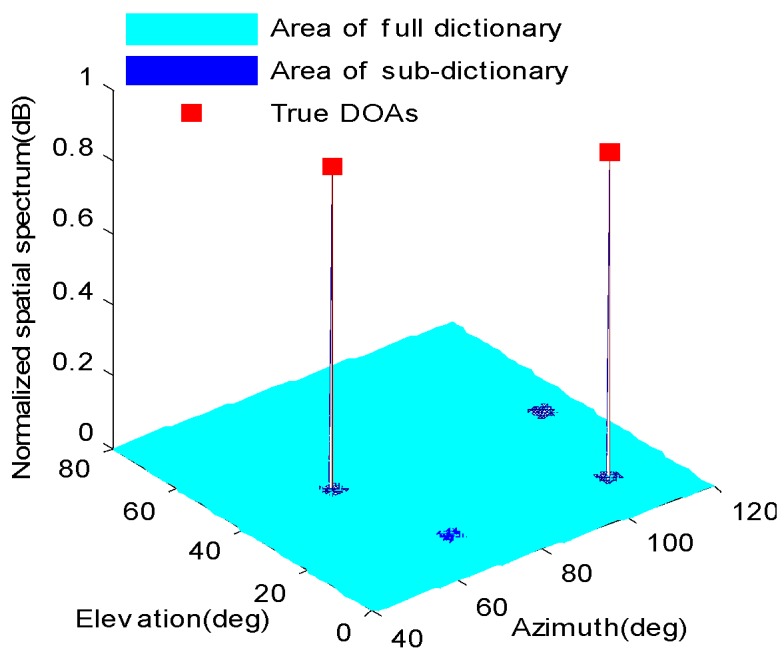
An example of pair-matching scheme based on sub-dictionary (two coherent sources).

Figure 2 shows the 2-D spatial spectrum obtained from Equation (36). It can be observed that only the true DOAs will produce spectrum peaks whereas there is no peak from the false sub-dictionaries. As a result, the pair-matching DOAs can be identified by checking whether there is a peak at the corresponding sub-dictionary. Furthermore, the 2-D angles of the *i*th source signal can be estimated by finding the central angles $\left({\hat{\theta }}^{i},{\hat{\varphi }}^{i}\right)$ of the *i*th sub-dictionary corresponding to the largest *K* peaks, that is

(38) $$\left({\hat{\theta }}^{i},{\hat{\varphi }}^{i}\right)=\arg\underset{i}{\max}\;Q\left(\theta ,\varphi \right)$$

Note that there is at most one source signal in each sub-dictionary, herein the selections of the radius and intervals only need to guarantee the sparsity of the spatial spectrum. Moreover, the accuracy and resolution requirements of the sparse solution in Equation (35) are relaxed so that greedy algorithm with low computation complexity, such as Orthogonal Matching Pursuit (OMP) [30] or Simultaneous OMP (SOMP) [31], can also be applied.

The proposed sub-dictionary based matching scheme is immune to amplitude estimation error. In addition, the SNR requirement is reduced. It raises the matching probability and applicable when the source powers are equal or close. The procedure of the proposed DRSR-SSRSD method can be summarized as follows:

Step 1: Construct the 1-D redundant dictionary of subarray $\Phi_{Z}$ and $\Phi_{X}$ according to Equations (19) and (20).
Step 2: Find the sparse solution ${\hat{S}}_{Z}$ and ${\hat{S}}_{X}$ according to Equation (26), then estimate the elevation angles $\left\{{\hat{\varphi }}_{1},{\hat{\varphi }}_{2},\cdots ,{\hat{\varphi }}_{K}\right\}$ and synthesis azimuth angles $\left\{{\hat{\alpha }}_{1},{\hat{\alpha }}_{2},\cdots ,{\hat{\alpha }}_{K}\right\}$ of K sources according to Equations (27) and (28).
Step 3: Calculate the corresponding azimuth angle ${\hat{\theta }}_{k}$ according to Equation (29) and form all combination Σ according to Equation (30).
Step 4: Angle matching. Construct 2-D sub-dictionary G according to Equations (31)–(34), then obtainits 2-D spatial spectrum according to Equations (36)–(38), and finally get the indexes of the sub-dictionary corresponding to the largest K spectrum peaks and their central angles $\left({\hat{\theta }}^{\;i},{\hat{\varphi }}^{i}\right)$ are the estimated DOAs.

## 5. Performance Analysis

### 5.1. Cramer–Rao Lower Bound

To facilitate derivation, Equations (15) and (16) are combined as

(39) $$W\left(t\right)=\left[\begin{array}{c} R_{Z}\left(t\right)\\ R_{X}\left(t\right) \end{array}\right]=\left[\begin{array}{c} A_{Z}S\left(t\right)+E_{Z}\left(t\right)\\ A_{X}S\left(t\right)+E_{X}\left(t\right) \end{array}\right]=\left[\begin{array}{c} A_{Z}\\ A_{X} \end{array}\right]S\left(t\right)+\left[\begin{array}{c} E_{Z}\left(t\right)\\ E_{X}\left(t\right) \end{array}\right]≜VS\left(t\right)+E\left(t\right)$$

Then the Cramer–Rao Lower Bound of the DOA estimation in Equation (39) is [18]

(40) CRB(ψ)=σN22J{Re[(DHPV⊥D)⊗SfT]}−1

where $\psi =\left(\theta^{T},\varphi^{T}\right)={\left(\theta_{1},\theta_{2},\cdots ,\theta_{K},\varphi_{1},\varphi_{2},\cdots ,\varphi_{K}\right)}^{T}$ is the 2-D DOA steering vector, $\sigma_{N}^{2}$ is noise power, J is number of snapshots, Re(⋅) denotes real part of the complex number, ⊗ represents Hadamard product, and D is derivation matrix as

(41) $$D=\frac{\partial V}{\partial \psi }=\left[\frac{\partial v_{1}}{\partial \theta_{1}},\frac{\partial v_{2}}{\partial \theta_{2}},\cdots ,\frac{\partial v_{K}}{\partial \theta_{K}},\frac{\partial v_{1}}{\partial \varphi_{1}},\frac{\partial v_{2}}{\partial \varphi_{2}},\cdots ,\frac{\partial v_{K}}{\partial \varphi_{K}}\right]$$

where $v_{i}$ is the *i*th column of matrix V, $P_{V}^{⊥}=I-V{\left(V^{H}V\right)}^{-1}V^{H}$ is the projection matrix of noise subspace, $S_{f}=E\left[SS^{H}\right]$ is the signal spectrum matrix, which is a diagonal matrix for uncorrelated signals as $S_{f}=\mathrm{diag}\left\{\sigma_{S,1}^{2},\sigma_{S,2}^{2},\cdots ,\sigma_{S,K}^{2}\right\}$ and a Hermite matrix for correlated signals written as

(42) $$S_{f}=\left[\begin{array}{cccc} \sigma_{S,1}^{2} & \rho_{12} & \ldots & \rho_{1K}\\ \rho_{12}^{*} & \sigma_{S,2}^{2} & \ldots & \rho_{2K}\\ \vdots & \vdots & \ddots & \vdots \\ \rho_{1K}^{*} & \rho_{2K}^{*} & \ldots & \sigma_{S,K}^{2} \end{array}\right]$$

where $\rho_{ij}$ is the correlation coefficient between the *i*th and the *j*th source signals.

### 5.2. Computational Complexity Analysis

The DRSR-SSRSD method adopts the idea of dimension reduction, which decomposes the 2-D search into two separate 1-D searches and followed by pair matching to facilitate 2-D DOA estimation. Herein, the computational complexity analyses involve 1-D DOA estimation algorithm and pair-matching algorithm.

● 1-D DOA estimation algorithm

The dimension of angle redundant dictionary Ψ is reduced from $L_{\theta }\times L_{\varphi }$ to $L_{\theta }$ or $L_{\varphi }$. Therefore, the computational complexity in 1-D DOA estimation by the L1-SVD [25] sparsity reconstruction is $O\left(K^{3}\left(L_{\theta }^{3}+L_{\varphi }^{3}\right)\right)$;

● Pair-matching algorithm

The pair-matching algorithm adopts the OMP or SOMP algorithm, which has low computational complexity as the undemanding reconstruction algorithm indicated in Section 4. The computational complexity of pair-matching algorithm is about $O\left(KJM_{c}L_{SD}\right)$ based on SOMP, where $L_{SD}$ stands for the number of the 2-D angles in the sub-dictionary.

Therefore, the total computational complexity is $O\left(K^{3}\left(L_{\theta }^{3}+L_{\varphi }^{3}\right)+KJM_{c}L_{SD}\right)$.

For comparison, three common sparse representation algorithms (i.e., OMP, BP and L1-SVD) are applied to DSR 2-D DOA estimation, and their computational complexity are listed in Table 1:

## 6. Simulation and Analysis

In the simulations, the cross array configuration is shown in Figure 1, in which the element number of each subarray is 11 and the array element spacing is half wavelength. The narrow-band coherent sources are generated by

(43) $$s_{i}\left(t\right)=\gamma_{i}e^{j\left(2\pi f_{0}t+\eta_{i}\right)}$$

where $\gamma_{i}$, $\eta_{i}$ and $f_{0}$ are the respective amplitude, phase and frequency of the *i*th signal. The center frequency is set to $f_{0}=300\;\text{MHz}$.

The SNR is defined as

(44) $$\mathrm{SNR}=10\log_{10}\left(\frac{1}{K}\sum_{k=1}^{K}\frac{\sigma_{S,k}^{2}}{\sigma_{N}^{2}}\right)$$

where $\sigma_{S,k}^{2}$ is the signal power of the *k*th signal and $\sigma_{N}^{2}$ is the noise power.

The Root Mean Square Error (RMSE) of the estimated DOA angles is defined as

(45) $$\mathrm{RMSE}=\sqrt{\frac{1}{2KN_{c}}\sum_{i=1}^{K}\sum_{j=1}^{N_{c}}\left[{\left({\hat{\theta }}_{i,j}-{\tilde{\theta }}_{i}\right)}^{2}+{\left({\hat{\varphi }}_{i,j}-{\tilde{\varphi }}_{i}\right)}^{2}\right]}$$

where $N_{c}$ is the number of independent Monte Carlo tests; and ${\tilde{\theta }}_{i}$ and ${\tilde{\varphi }}_{i}$ are the real azimuth and elevation angles of the *i*th signal, respectively, while ${\hat{\theta }}_{i}$ and ${\hat{\varphi }}_{i}$ are their estimated values.

### 6.1. DOA Estimated under Identical Source Amplitudes

**Figure 3:**
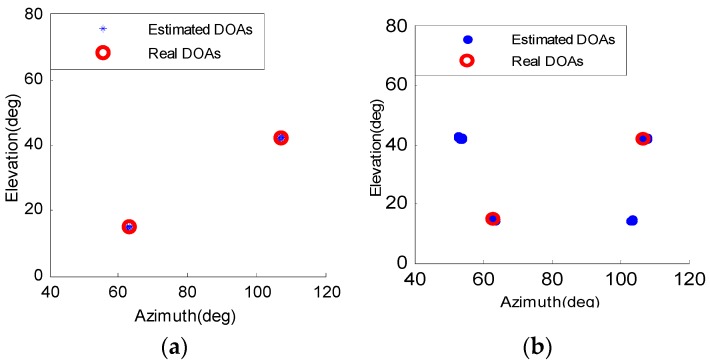

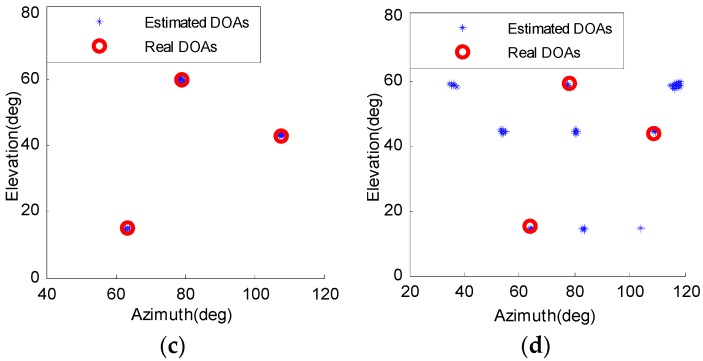
2-D DOA estimation results of DRSR-SSRSD and SRSA-AC method: (**a**) DRSR-SSRSD (two coherent signals); (**b**) SRSA-AC (two coherent signals); (**c**) DRSR-SSRSD (three coherent signals); and (**d**) SRSA-AC (three coherent signals).

Figure 3 illustrates the 2-D DOA estimated result of two and three coherent signals with same amplitude for 100 independent Monte Carlo tests. The SNR is 0 dB and the number of snapshot is 50. The azimuth and elevation of the two coherent signals in Figure 3a,b are $\left[\theta_{1},\varphi_{1}]=[63^{o},15^{o}\right]$ and $\left[\theta_{2},\varphi_{2}]=[107^{o},42^{o}\right]$. The three coherent signals in Figure 3c,d are set to be $\left[\theta_{1},\varphi_{1}]=[63^{o},15^{o}\right]$, $\left[\theta_{2},\varphi_{2}]=[107^{o},42^{o}\right]$ and $\left[\theta_{3},\varphi_{3}]=[80^{o},60^{o}\right]$. It is observed that the proposed DRSR-SSRSD method can pair match correctly, while SRSA-AC [27] method fails.

### 6.2. Performance Analysis under Different SNRs

**Figure 4:**
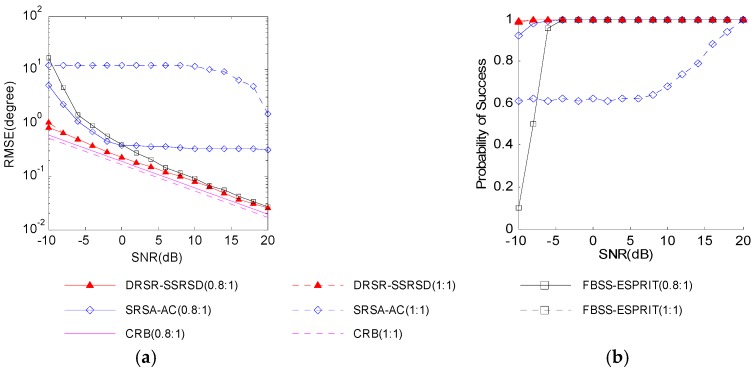
Estimation performance versus SNRs: (**a**) estimation error; and (**b**) pair-matching probability.

Figure 4 illustrates that the 2-D DOA estimation error and pair-matching probability vary with SNRs under amplitude ratios of $\gamma_{1}/\gamma_{2}=1:1$ and $\gamma_{1}/\gamma_{2}=0.8:1$ of two coherent signals. The azimuth and elevation angles are set as $\left[\theta_{1},\varphi_{1}]=[52^{o},25^{o}\right]$ and $\left[\theta_{2},\varphi_{2}]=[96^{o},72^{o}\right]$, respectively, and the number of snapshots is 100.

Figure 4 shows that proposed DRSR-SSRSD method performs well regardless of whether the amplitudes of the signals are identical, whereas for SRSA-AC method the pair-matching failure leads to a poor angle estimation. The estimation error remains close to the CRB at low SNRs (−10 dB~0 dB). The estimation performance of FBSS-ESPRIT [11] method is not affected by the amplitude ratio, but it will get worse under lower SNRs.

### 6.3. Performance Analysis under Different Amplitudes

**Figure 5:**
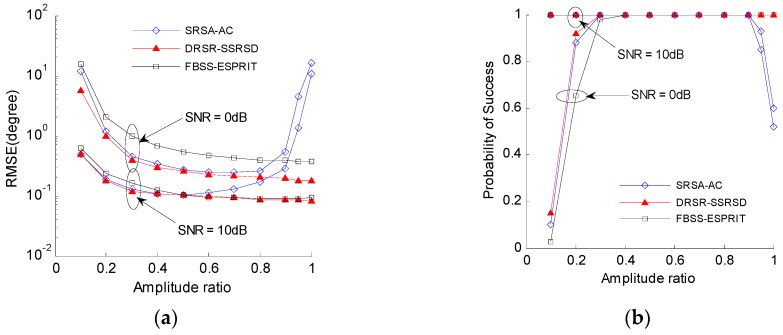
Estimation performance versus amplitude ratio: (**a**) estimation error; and (**b**) pair-matching probability.

Figure 5 illustrates that the 2-D DOA estimation error and pair-matching probability vary with different amplitude ratios of $\gamma_{1}/\gamma_{2}$ of two coherent signals. The SNR is 0 dB and the number of snapshots is 100.

Simulation results show that the estimation performance of proposed DRSR-SSRSD method becomes better with the increase of amplitude ratio. In contrast, the estimation performance of SRSA-AC method deteriorates when the amplitude ratio is larger than 0.5, especially when the amplitude ratio is larger than 0.9, the performance drops significantly. The FBSS-ESPRIT method has the similar performance to DRSR-SSRSD method when SNR is 10 dB, whereas when SNR drops to 0 dB, the performance of FBSS-ESPRIT method is much worse than that of DRSR-SSRSD method.

### 6.4. RMSE Analysis in Different Number of Snapshots

**Figure 6:**
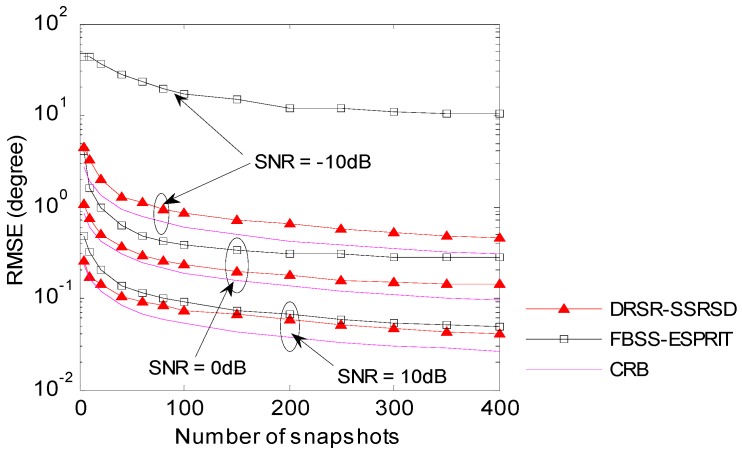
Estimation RMSE versus Snapshots under different SNRs.

Figure 6 illustrates the relationship between 2-D DOA estimation error and the number of snapshots when the SNRs are −10 dB, 0 dB and 10 dB. Two coherent signals are with equal amplitude and from same incident angles in Section 6.2. The RMSE performance of DRSR-SSRSD method is close to the CRB under different SNRs and different number of snapshots, while FBSS-ESPRIT method generates a poor estimation output in low SNR and its performance is not improved much even if the number of snapshots increases. In addition, it is worth noting that proposed DRSR-SSRSD method still performs well under a single snapshot condition.

### 6.5. RMSE Analysis in Different Angle Intervals

**Figure 7:**
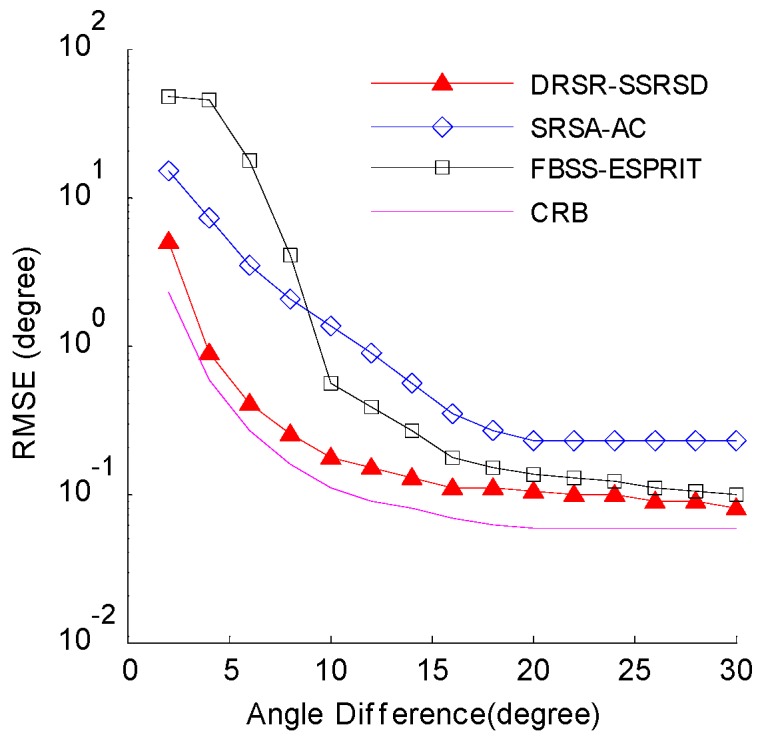
Estimation RMSE versus Angle intervals.

Figure 7 illustrates the relationship between 2-D DOA estimation error and different angle intervals when the amplitude ratio of the coherent signals is 0.8:1. The SNR is 10 dB and the number of snapshots is 100. In Figure 7, the performance of SRSA-AC method is better than that of FBSS-ESPRIT method when the angle interval is less than 10 degrees, while FBSS-ESPRIT performs better than SRSA-AC otherwise. The proposed DRSR-SSRSD method gives the smallest estimation error.

### 6.6. RMSE Analysis under Different Correlation Coefficients

**Figure 8:**
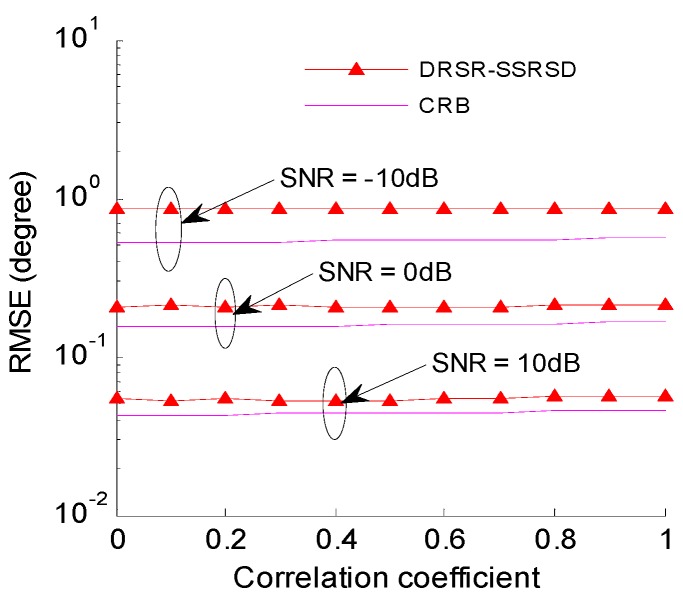
Estimation RMSE versus Correlation coefficient under different SNRs.

Figure 8 illustrates the relationship between 2-D DOA estimation error and different correlation coefficients when the SNRs are −10 dB, 0 dB and 10 dB. The signals with different correlation coefficients are [8]

(46) $$s_{2}\left(t\right)=\rho s_{1}\left(t\right)+\sqrt{1-{\left|\rho \right|}^{2}}s_{2o}\left(t\right)$$

where $s_{1}\left(t\right)$ and $s_{2o}\left(t\right)$ are two independent signals with identical power, and ρ is the correlation coefficient of $s_{1}\left(t\right)$ and $s_{2}\left(t\right)$ which ranges from 0 to 1. Those correlated signals have the same power.

Simulation results show that the RMSE performance of DRSR-SSRSD method does not change with the increase of the correlation coefficients even under low SNR. Therefore, the high precision 2-D coherent DOA estimation is achieved.

### 6.7. RMSE Analysis of Uniform T-Shaped, l-Shaped and Cross Arrays

**Figure 9:**
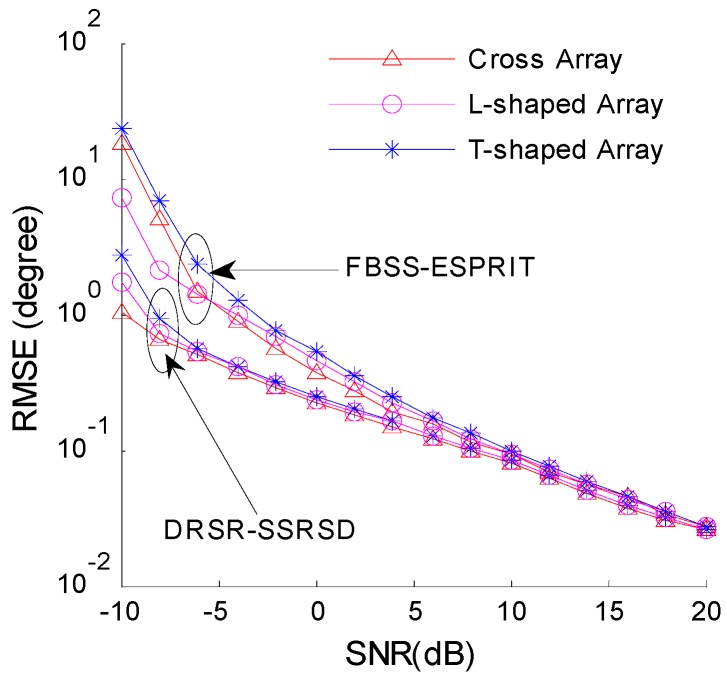
Estimation RMSE versus SNRs in different array configurations.

Figure 9 illustrates the 2-D DOA estimation error of DRSR-SSRSD and FBSS-ESPRIT method of T-shaped, l-shaped and cross arrays. The number of array elements is identical for all three array formations whose array elements are uniformly spaced with half wavelength. It is observed that the estimation performance of DRSR-SSRSD under the three types of orthogonal array is almost the same, especially in the high SNR condition. Overall, the performance of DRSR-SSRSD is superior to FBSS-ESPRIT.

### 6.8. Computational Complexity Analysis

**Figure 10:**
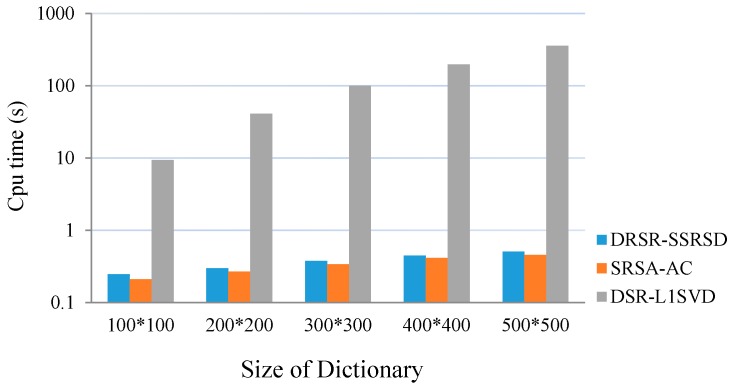
Computation time of three methods.

Figure 10 illustrates the 2-D DOA estimation computation time of DRSR-SSRSD, SRSA-AC and DSR-L1SVD. Assuming that there are two coherent signals, SNR is 10 dB and the number of snapshots is 100.

Figure 10 shows that the computation time of DRSR-SSRSD method decreases significantly compared with the DSR-L1SVD method, while slightly higher than SRSA-AC method due of the additional pairing. From the theoretical analysis and simulation results, the computational complexity brought by the pairing algorithm is comparatively a small proportion of the whole method. Especially, the larger the size of dictionary is, the smaller the proportion is. Therefore, compared with SRSA-AC method, the estimation performance of DRSR-SSRSD method is improved by slightly scarifying the computational complexity.

## 7. Conclusions

A novel DOA estimation method based on dimension reduction sparse reconstruction and Spatial Spectrum Reconstruction of Sub-Dictionary (DRSR-SSRSD) has been proposed to realize a low complexity 2-D coherent DOA estimation. Meanwhile, the problem that the pairing algorithm failed when the signals’ power is equal or close has been solved successfully. Simulation results demonstrate that the proposed method can facilitate the coherent DOA estimation with almost the same performance in different coherent coefficients. The estimation error approaches CRB even under the conditions of small snapshot number, low SNR and small angle interval. The proposed method has lower complexity compared with direct sparse representation methods. Moreover, its application can be extended to any uniform and non-uniform orthogonal arrays without a priori knowledge of exact signal number.

## Figures and Tables

**Table 1:** Complexity comparison with common sparse decomposition algorithms.

Algorithms	Single-Snapshot Case	Multiple Snapshots Case
DSR-OMP	$O\left(KM_{c}L_{\theta }L_{\varphi }\right)$	$O\left(KJM_{c}L_{\theta }L_{\varphi }\right)$
DSR-BP	$O\left({\left(L_{\theta }L_{\varphi }\right)}^{3}\log\left(L_{\theta }L_{\varphi }\right)\right)$	$O\left({\left(JL_{\theta }L_{\varphi }\right)}^{3}\log\left(JL_{\theta }L_{\varphi }\right)\right)$
DSR-L1SVD	$O\left({\left(L_{\theta }L_{\varphi }\right)}^{3}\right)$	$O\left(K^{3}{\left(L_{\theta }L_{\varphi }\right)}^{3}\right)$
DRSR-SSRSD	$O\left(L_{\theta }^{3}+L_{\varphi }^{3}+KM_{c}L_{SD}\right)$	$O\left(K^{3}\left(L_{\theta }^{3}+L_{\varphi }^{3}\right)+KJM_{c}L_{SD}\right)$
